# Integrating Choline and Specific Intestinal Microbiota to Classify Type 2 Diabetes in Adults: A Machine Learning Based Metagenomics Study

**DOI:** 10.3389/fendo.2022.906310

**Published:** 2022-06-27

**Authors:** Qiang Zeng, Mingming Zhao, Fei Wang, Yanping Li, Huimin Li, Jianqiong Zheng, Xianyang Chen, Xiaolan Zhao, Liang Ji, Xiangyang Gao, Changjie Liu, Yu Wang, Si Cheng, Jie Xu, Bing Pan, Jing Sun, Yongli Li, Dongfang Li, Yuan He, Lemin Zheng

**Affiliations:** ^1^ Health Management Institute, the Second Medical Center & National Clinical Research Center for Geriatric Diseases, Chinese People's Liberation Army General Hospital, Beijing, China; ^2^ China National Clinical Research Center for Neurological Diseases, Tiantan Hospital, Advanced Innovation Center for Human Brain Protection, The Capital Medical University, Beijing, China; ^3^ The Institute of Cardiovascular Sciences and Institute of Systems Biomedicine, School of Basic Medical Sciences, Peking University Health Science Center, Key Laboratory of Molecular Cardiovascular Science of Ministry of Education, Key Laboratory of Cardiovascular Molecular Biology and Regulatory Peptides of Ministry of Health, Beijing Key Laboratory of Cardiovascular Receptors Research, Beijing, China; ^4^ Department of Nutrition, Harvard T.H. Chan School of Public Health, Boston, MA, China; ^5^ National Human Genetic Resources Center, Beijing, China, National Research Institute for Family Planning, Beijing, China; ^6^ Chinese Academy of Medical Sciences & Peking Union Medical College, Beijing, China; ^7^ Department of Obstetrics and Gynecology, The Third Clinical Institute Affiliated to Wenzhou Medical University, The Third Affiliated Hospital of Shanghai University, Wenzhou People’s Hospital, Wenzhou Maternal and Child Health Care Hospital, Wenzhou, China; ^8^ Bao Feng Key Laboratory of Genetics and Metabolism, Zhongyuan Biotechnology Holdings Group, Beijing, China; ^9^ Zhong Guan Cun Biological and Medical Big Data Center, Zhong Guan Cun Medical Engineering & Health Industry Base, Beijing, China; ^10^ Southwest Hospital, Third Military Medical University, Chongqing, China; ^11^ Health Management Center, The 910th Hospital of People's Liberation Army, Quanzhou, China; ^12^ Health Management Center, The China-Japan Union Hospital of Jilin University, Changchun, China; ^13^ Department of Health Management, Henan Provincial People’s Hospital, Zhengzhou, China; ^14^ Department of Microbial Research, WeHealthGene Institute, Shenzhen, Guangdong, China; ^15^ Institute of Statistics, NanKai University, Tianjin, China

**Keywords:** choline, intestinal microbiota, TMAO, type 2 diabetes, machine learning

## Abstract

Emerging evidence is examining the precise role of intestinal microbiota in the pathogenesis of type 2 diabetes. The aim of this study was to investigate the association of intestinal microbiota and microbiota-generated metabolites with glucose metabolism systematically in a large cross-sectional study in China. 1160 subjects were divided into three groups based on their glucose level: normal glucose group (n=504), prediabetes group (n=394), and diabetes group (n=262). Plasma concentrations of TMAO, choline, betaine, and carnitine were measured. Intestinal microbiota was measured in a subgroup of 161 controls, 144 prediabetes and 56 diabetes by using metagenomics sequencing. We identified that plasma choline [Per SD of log-transformed change: odds ratio 1.36 (95 confidence interval 1.16, 1.58)] was positively, while betaine [0.77 (0.66, 0.89)] was negatively associated with diabetes, independently of TMAO. Individuals with diabetes could be accurately distinguished from controls by integrating data on choline, and certain microbiota species, as well as traditional risk factors (AUC=0.971). KOs associated with the carbohydrate metabolism pathway were enhanced in individuals with high choline level. The functional shift in the carbohydrate metabolism pathway in high choline group was driven by species *Ruminococcus lactaris*, *Coprococcus catus* and *Prevotella copri*. We demonstrated the potential ability for classifying diabetic population by choline and specific species, and provided a novel insight of choline metabolism linking the microbiota to impaired glucose metabolism and diabetes.

## 1 Introduction

Recently, emerging evidence is examining the precise role of intestinal microbiota in the pathogenesis of type 2 diabetes (diabetes) (1). Data are accumulating that patients with diabetes had a moderate intestinal dysbiosis. Metagenome-wide association studies have demonstrated a highly significant association between butyrate-producing bacteria such as *Roseburia intestinalis* and *Faecalibacterium prausnitzii* concentrations and diabetes (2). Fecal transplantation in humans further highlights the possibility of modulating human metabolism by directly altering the microbiota, showing that insulin sensitivity was improved along with the increase of butyrate-producing bacteria after fecal transferring from lean donors to male recipients with metabolic syndrome (3). Microbiota may directly modulate host metabolism by short-chain fatty acids especially butyrate, endotoxaemia, and specific intestinal bacteria (such as *Akkermansia muciniphila*) which plays a role in anti-inflammatory and beneficial metabolic functions (4).

Trimethylamine-N-oxide (TMAO) is a plasma metabolite and its generation is dependent on the intestinal microbiota from TMA, which primarily metabolizes from dietary choline, betaine and L-carnitine in the intestinal tract. Thereafter, TMA is metabolized to TMAO by enzymes of the flavin monooxygenase (FMO) family in liver (5). Numerous studies have demonstrated TMAO is a novel predictive risk factor of adverse cardiovascular outcomes (5–7). The mechanism appears to involve that TMAO interacting with platelets, altering stimulus-dependent calcium signaling, fostering platelet hyper-reactivity *in vivo*, and promoting vascular inflammation in animal models (8). Several studies further demonstrated that plasma TMAO was elevated in patients with diabetes compared to healthy controls, possibly due to TMAO converting enzyme FMO3 which exerted broad effects on glucose and lipid metabolism (9). Knockdown of hepatic FMO3 significant decreased circulating TMAO levels and atherosclerosis in mice, accompanying decreases in hepatic lipids and in levels of plasma lipids, glucose, and insulin (10). Also, recent study showed that elevated levels of circulating choline were significantly associated with diabetes (11). Intervention study has found the associations between change in choline and that in insulin sensitivity independently of concurrent changes in TMAO (12). However, advanced correlations of blood glucose, related phenotypes and microbial metabolites including plasma choline, betaine and TMAO, and whether these metabolites are related to human intestinal microbiota were unknown. Thus, the aim of this study was to investigate the associations of TMAO and its precursors (choline, betaine and carnitine) with glucose metabolism, and to explore the potential mechanism targeting intestinal microbiota and their effects on the human health.

## 2 Methods

### 2.1 Population

**Figure 1:**
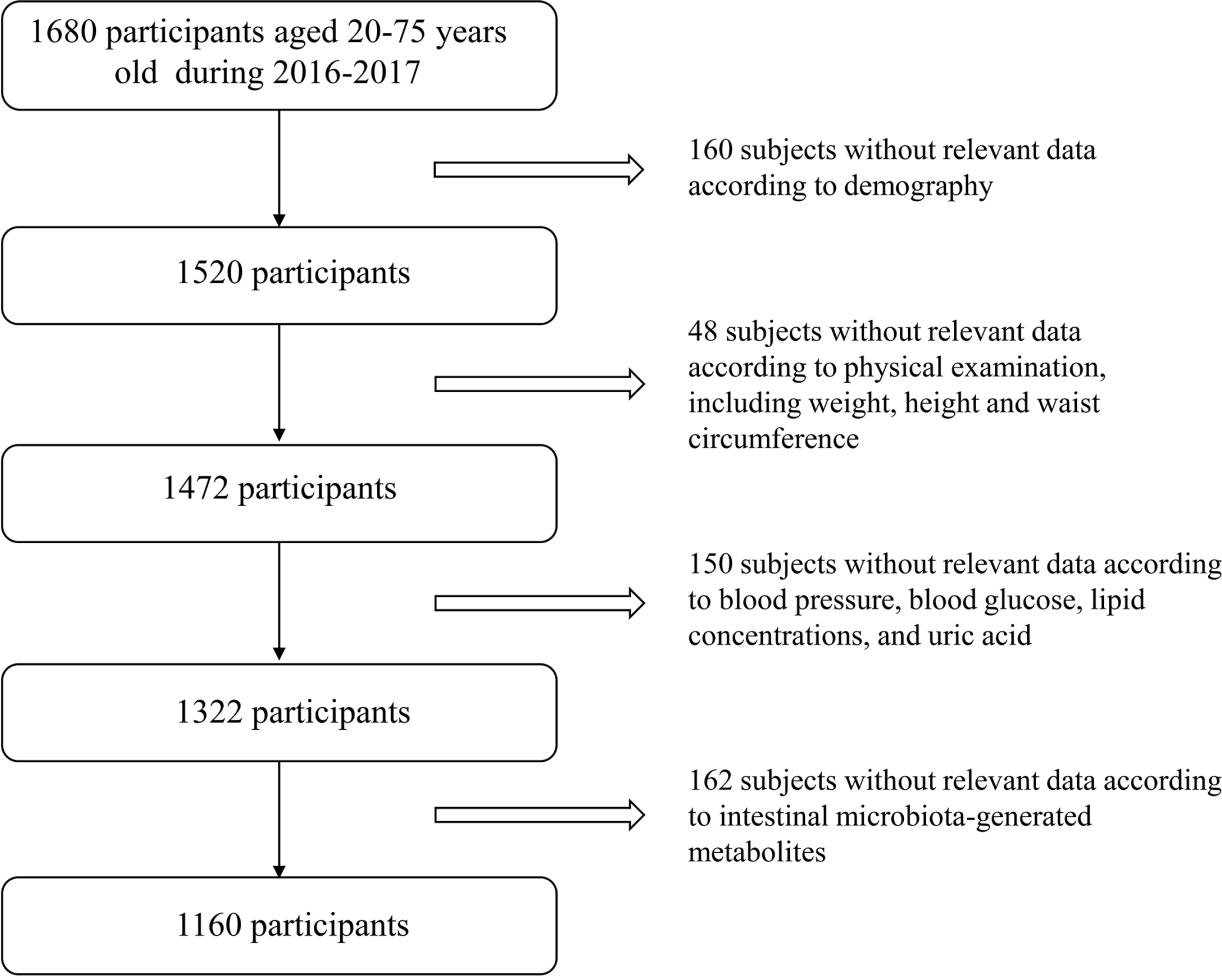
Flow chart of the study participant.

We conducted a cross-sectional study at three health examination centers from Jan. 2016 to Sep. 2017, aiming to examine the association of intestinal microbiota, microbiota-generated metabolites with glucose metabolism in Chinese adults. The two-stage cluster sampling method was used to, first selected three cities according to geographical region and dietary/lifestyle habit (Northern region: Changchun; Southern region: Quanzhou; Western region: Chongqing), and then selected one local representative health examination center from each city (Changchun: The China-Japan Union Hospital; Quanzhou: The 910th Hospital of People’s Liberation Army; Chongqing: Southwest Hospital). A total of 1160 subjects (aged 20-75 years) who participated in annual health examinations were randomly selected in each center with complete information on demographics, personal characteristics (including weight, height and waist circumference) and clinical characteristics (including blood pressure, blood glucose, lipid concentrations, uric acid and serum creatinine). Exclusion criteria for study participation included: i) younger than 20 years or older than 75 years; ii) exposed to antibiotic, probiotics, acid reducing medications or proton pump inhibitor one month before physical examination; iii) suffered from diarrhea, constipation, hematochezia or other gastrointestinal infectious diseases one month prior to physical examination; iv) experienced enema or other gastroenterology operations one month before physical examination; v) suffered from mental disorders, autoimmune diseases or psychological imbalance; vi) had drug abuse history, which resulted in 1160 subjects for current study. Detailed study flow is shown in **Figure 1**. This study was approved by the Ethical Committee of the Chinese People’s Liberation Army General Hospital and was in accordance with the Helsinki Declaration. Every subject provided written informed consent.

### 2.2 Outcomes

Type 2 Diabetes status was used as main outcomes. Prediabetes was defined as a fasting plasma glucose from 5.6 to 6.9mmol/L, and diabetes was defined as a fasting plasma glucose ≥7.0mmol/L. In addition, the following outcomes were used: hypertension was defined as having a systolic blood pressure ≥140mmHg and/or diastolic blood pressure ≥90mmHg; dyslipidemia was defined as having plasma total cholesterol ≥6.22mmol/L and/or fasting triglycerides ≥2.26mmol/L and/or LDL cholesterol ≥4.14mmol/L and/or HDL-cholesterol <1.04mmol/L; hyperuricemia was defined as having uric acid ≥420 mg/dL for men and ≥357mg/dL for women.

### 2.3 Covariates

All clinical data was collected according to standard procedures. Subjects underwent anthropometric measurements in barefoot and light clothing. Body weight (measured to the nearest 0.1kg) and height (measured to the nearest 0.1cm) were collected and BMI was calculated by dividing weight (kg) by height squared (m2). Blood pressure was recorded using a recently calibrated electronic sphygmomanometer in the supine position with the right arm after 5minutes rest. Blood samples were obtained after an overnight fast for measurement of blood glucose, total and high-density lipoprotein cholesterol, triglycerides, uric acid and serum creatinine. Low-density lipoprotein cholesterol was calculated using the Friedewald formula. Serum creatinine was measured using Jaffe’s kinetic method. All blood samples were analyzed at a local laboratory in each city rather than a central laboratory. Because all the laboratories were affiliated with a top tertiary hospital and completed a standardized and certificated method for blood test, these results have been widely considered comparably across laboratories in China.

### 2.4 Microbiota-Generated Metabolites Measurements

Analytes (TMAO, betaine, choline, and carnitine) were measured in one center laboratory as described previously (13). Briefly, 20μl plasma were mixed with 80μl of 10μM d9-(trimethyl)-labelled internal standards in methanol. Protein was precipitated and the supernatant was recovered following centrifugation at 20,000g at 4°C for 10min. The precise concentration was measured by API 5500Q-TRAP mass spectrometer (AB SCIEX, Framingham, MA). Analytes were monitored using electrospray ionization in positive-ion mode with multiple reaction monitoring (MRM) of precursor and characteristic product-ion transitions of TMAO at m/z 76→58, d9-TMAO at m/z 85→66, choline at m/z 104→59.8, d9-choline at m/z 113.2→68.9, carnitine at m/z 162.1→103, d9-carnitine at m/z 171.1→102.8, betaine at m/z 118→59, d11-betaine at 129.1→65.9, respectively. Three quality control samples with different metabolites concentrations were measured every twenty samples and the CV% values were below 10%. The quartiles based on TMAO, choline, betaine, and carnitine, separately, TMAO levels for the quartile groups were as follows: Q1 <0.98, Q2: 0.98~1.58, Q3: 1.59~2.52, Q4 > 2.52mmol/L; Choline levels for the quartiles were as follows: Q1 <7.2, Q2: 7.2~8.5, Q3: 8.6~10.1, Q4 > 10.1mmol/L; Betaine levels for the quartiles were as follows: Q1 < 37.3, Q2: 37.3~43.3, Q3: 43.4~51.3, Q4 > 51.3mmol/L. Carnitine levels for the quartiles were as follows: Q1 < 48.2, Q2: 48.2~55.1, Q3: 55.2~61.9, Q4 > 61.9mmol/L.

### 2.5 Gut Microbiota Measurements

#### 2.5.1 Stool Sample Collection and DNA Extraction

During physical examination, fresh stools were collected from the individuals using sterile stool containers. For each individual, approximately 5g of hard stools were obtained using the swab (Huachenyang Technology CO., LTD, Shenzhen, China). The stool samples were preserved using stool collection tubes (Axygen, California, USA) with Microlution (ML001-A, Dayun Ltd, Shenzhen, China), and then transferred to -80°C refrigerator (DW-86L626, Haier, China) within half an hour. Bacterial DNA was extracted from stool samples using Power Soil DNA Isolation kit (Mo Bio Laboratories, Carlsbad) at WeHealthGene Co., Ltd according to the manufacturer’s instruction.

#### 2.5.2 Library Construction and Metagenomics Sequencing

DNA library construction was performed with the following workflow as suggested by the manufacturer (Illumina, San Diego): cluster generation, template hybridization, isothermal amplification, linearization, blocking and denaturation, and hybridization of the sequencing primers. We constructed paired-end (PE) library with insert size of 350bp, and each sample contains around 20 million PE reads after high-throughput sequencing. For samples at stages I, their libraries were sequenced with 75 or 90 base pairs, while the libraries were sequenced with 90 base pairs for samples at stage II.

#### 2.5.3 Quality Control and Host Genome Filtering

High quality reads were obtained with the following filtering criteria: If any one of paired-end reads i) contains10% ambiguous N bases; ii) or more than 50% low quality (Q<5) bases, the paired-end reads were thrown away. Then, the clean reads were subjected to human genomes (human genome reference hg19) from the National Center for Biotechnology Information GenBank with SOAPaligner (version 2.21,”-m 250 -x 450 -v 5 -r 1 -l 35 -M 4”), and the reads which mapped to human genome were abandoned (14). The filtered reads were retained for further analysis.

#### 2.5.4 Gene Abundance, Functional Annotation and Taxonomic Profiling

The qualified reads from the samples were aligned to the upgraded non-redundant gene catalogue (15) with SOAPaligner (version 2.21, “-m 250 -x 450 -v 5 -r 1 -l 35 -M 4”), wand the mapped reads with less than 7 mismatches were kept. Based on the gene length and the number of mapped reads, the abundances of genes were obtained for each sample with previous published method (16).

To obtain the functional distributions of genes, we aligned them to the proteins/domains in KEGG databases (release 59.0) and CAZy database using BLASTP (e-value ≤1e-5). The KEGG orthologue group (KO) or CAZy families with the highest scoring annotated hit(s) which containing at least one HSP (high-scoring segment pair) scoring over 60 bits was selected. The abundance of KEGG orthology/module in each sample was calculated by summing the abundance of genes which annotated to the same functional item. With shotgun metagenomic data, the composition of microbial community on different taxonomic level was detected for each sample using MetaPhlAn2 pipeline with default parameters (17).

### 2.6 Statistical Analyses

Continuous variables are summarized as mean (SD) if normally distributed and median [interquartile range (IQR)] if nonnormally distributed. The unpaired Student t-test or Wilcoxon signed rank test for continuous variables and Chi-squared test for categorical variables were employed to examine between group differences. The associations between intestinal microbiota-generated metabolites and diabetes were examined by applying logistic regression models with adjustment for potential confounders including age, sex and BMI. The levels of TMAO, betaine, choline, carnitine were divided into quartiles and the lowest quartile was used as the reference group. Sensitivity analysis was conducted i) by including lifestyle factors, alcohol consumption, smoking habit, dietary habit, exercise habit, sleeping habit, stool shape, whether eating probiotics supplements, whether having conditions of regular defecation, diarrhea, or constipation, as covariates in a subgroup population; ii) by further adjusted other metabolites in the models. Statistical analysis was performed using STATA software version 13.0 (StataCorp., College Station, TX) or GraphPad Prism 6 software. Statistical tests were 2-sided and a P value<0.05 was considered statistically significant.

We pre-processed the intestinal microbiota abundant data and deleted the variables with 0 value greater than 20%. The Shannon index and principal coordinates analysis (PCoA) was calculated with the vegan package in R software (Version 3.4.3). PCoA was performed and displayed by ade4 package, cluster packages, fpc packages, and clusterSim package in R software. PLS-DA was performed using SIMCA-P software to cluster the sample plots across groups. The relative abundance of these features was subjected to statistical analyses. Linear discriminant analysis (LDA) effect size (LEfSe) analysis was used to detect the features (organisms, KOs, or CAZy genes) most likely to explain differences between the prediabetes, diabetes and control group, as well as high (top quartile) and low groups (lowest quartile) of choline and TMAO. Different features with an LDA score cut-off of 2.0 were identified. Taxa-based functional profiles was calculating by FishTaco software. Correlations between enriched species, metabolites and clinical indices were tested with MaAslin2. Dimension reduction analysis was based on the PLS-DA, where the variables were selected by variable importance projection (VIP)>1 and mean difference screening (P<0.05) as biomarkers 1; variables were selected by one-way ANOVA (P<0.05) as biomarkers 2; only microbiota indicators were selected by one-way ANOVA (P<0.05) as biomarkers 3; and traditional risk factors were selected as biomarkers 4. Classification machine learning algorithms using Support Vector Machines (SVM), Random Forests (RF), Decision Tree (DT) were performed to obtain the optimal diagnostic model using R. The OPLS-DA model analysis was based on muma and ropls package, and the SVM, RF and DT and was based on svm, random forest, and rpart package. Then, in order to evaluate the performance of the predictive model and get more precise curves, we used a 10-fold cross-validation for each model. ROC curve analysis was performed using the highest validated AUC values, and variable importance was measured by GINI coefficient. The ROC curves were conducted by pROC package.

## 3 Results

### 3.1 Association of Intestinal Microbiota-Generated Metabolites With Prediabetes and Diabetes

We conducted a cross-sectional study including a total of 1160 subjects (aged 20-75 years) who participated in annual health examinations. The sample size varied according to the number of missing data, with missing data on metabolites outcome variables (n =1 for TMAO and choline, as well as n =5 for betaine), or other covariates (n =33). Baseline characteristics of the 1160 participants are shown in **Table 1**. The median (mean) plasma concentrations of TMAO, choline, betaine, and L-carnitine were 1.59 μmol/L (IQR: 0.98 to 2.52μmol/L), 8.58 μmol/L (IQR: 7.23 to 10.10μmol/L), 43.4 μmol/L (IQR: 37.30 to 51.32 μmol/L), and 55.2 ± 10.8 (mean ± SEM), respectively. Participants with higher levels of blood glucose were more likely to be older, had a higher proportion of males, and had higher levels of BMI, blood pressure, and were more likely to be dyslipidemia and hypeluricemia. In the three groups of diabetes, prediabetes and controls, TMAO was significantly higher in participants with diabetes compared with controls, and choline was higher in participants with hyperglycemia than controls. There was an inverse dose-response association between plasma betaine concentration and fasting glucose in the three groups. Cubic spline curves showed that TMAO associated with blood glucose as a J-shape. Choline linearly increased with increasing blood glucose, while betaine linearly decreased with increasing blood glucose (**Figure S1**)

**Table 1:** Baseline characteristics according to controls, prediabetes and diabetes.

	Totaln = 1160	Controlsn = 504	Prediabetesn = 394	Diabetesn = 262	P value
Age, years	46.0 (38.0, 52.0)	42.0 (35.0, 51.0)	47.0 (40.0, 52.0)	48.0 (42.0, 53.0)	< 0.001
Male, %	65.2	57.9	68.0	74.8	< 0.001
BMI, kg/m^2^	27.4 (25.4, 29.3)	27.5 (24.7, 29.4)	27.5 (26.0, 29.0)	28.2 (26.4, 30.9)	< 0.001
Systolic BP, mmHg	129 (118, 139)	125 (114, 135)	129 (119, 140)	135 (123, 146)	< 0.001
Diastolic BP, mmHg	82 ± 12	79.6 ± 11.4	83.0 ± 12.5	85.0 ± 12.6	< 0.001
Fasting glucose, mmol/L	5.7 (5.2, 6.6)	5.2 (4.9, 5.4)	5.9 (5.7, 6.1)	8.5 (7.3, 10.5)	< 0.001
Total cholesterol, mmol/L	5.3 (4.6, 5.9)	5.0 (4.4, 5.7)	5.4 (4.8, 5.9)	5.4 (4.9, 6.2)	< 0.001
Triglycerides, mmol/L	1.8 (1.2, 2.7)	1.5 (1.0, 2.3)	1.8 (1.3, 2.6)	2.4 (1.6, 3.7)	< 0.001
HDL cholesterol, mmol/L	1.2 (1.1, 1.4)	1.2 (1.1, 1.4)	1.2 (1.1, 1.4)	1.2 (1.0, 1.4)	0.082
LDL cholesterol, mmol/L	2.8 (2.4, 3.2)	2.7 (2.3, 3.2)	2.8 (2.4, 3.2)	2.9 (2.5, 3.5)	< 0.001
Uric acid, mg/dL	372.5 ± 98.0	360.5 ± 100.1	387.6 ± 97.4	373.2 ± 92.0	< 0.001
Hypertension, %	33.7	25.4	35.8	46.6	< 0.001
Dyslipidemia, %	52.2	42.3	52.0	71.4	< 0.001
Hypeluricemia, %	34.4	29.8	39.8	37.0	< 0.001
TMAO, μmol/L	1.59 (0.98, 2.52)	1.5 (0.9, 2.3)	1.6 (1.0, 2.5)	1.7 (1.1, 3.3)	0.040
Choline, μmol/L	8.58 (7.23, 10.10)	8.2 (6.9, 9.9)	8.3 (7.1, 9.8)	9.3 (7.9, 11.0)	< 0.001
Betaine, μmol/L	43.4 (37.30, 51.32)	44.4 (37.7, 52.9)	43.6 (37.7, 51.2)	41.0 (35.2, 48.6)	0.003
Carnitine, μmol/L	55.2 ± 10.8	55.5 ± 10.4	55.4 ± 10.8	54.5 ± 11.7	0.434

BMI, body mass index; BP, blood pressure; HDL, high-density lipoproterin; LDL, low-density lipoprotein; TMAO, trimethylamine N-oxide.

Following multivariate logistic regression analyses adjusting for age, sex and BMI, each SD increment in log-transformed plasma concentration for TMAO and choline was associated with 16-36% increased odds of diabetes (*P*<0.05), while each SD increment of log-transformed plasma betaine was correlated with 23% decreased odds of diabetes (*P*<0.001) (**Table 2**). Participants in the top quartile of TMAO had 1.67 fold higher odds of diabetes compared with the lowest quartile. In general, the association of plasma TMAO, choline and betaine with diabetes was consistent across total group, and subgroups after stratification by sex and age groups (all *Ps* for interaction > 0.05). In the sensitivity analyses, the odds ratios for the metabolites did not change appreciably with additional adjustment for lifestyle factors in the subgroup; Findings were similar when per SD of all metabolites were included into the same adjusted model, that TMAO [odds ratio and 95%CI: 1.25 (1.01-1.54)], choline [1.74 (1.38-2.19)], betaine [0.58 (0.47-0.71)] and carnitine [0.72 (0.59-0.89)] remained significantly associated with diabetes. Parameter estimates were slightly attenuated after further adjustment for metabolic biomarkers including blood pressure, lipids and uric acid. Further, when we put four quartiles of all metabolites into one model, the association between the top quartile of TMAO and diabetes was also significant (1.57 [1.03-2.39]).

**Table 2:** Relationship between plasma concentrations of TMAO, choline, betaine, carnitine and diabetes (μmol/L)^a^.

	Per SD of log-transformed change^*^	P value	Quartiles^*^				P for trend
			1 (lowest)	2	3	4 (highest)	
**Total**							
TMAO	1.16 (1.00, 1.35)	0.049	1.00	1.14 (0.75, 1.73)	0.92 (0.60, 1.41)	1.67 (1.11, 2.51)^*^	0.035
Choline	1.36 (1.16, 1.58)	< 0.001	1.00	1.32 (0.86, 2.05)	1.68 (1.10, 2.57)^*^	1.93 (1.27, 2.92)^**^	0.001
Betaine	0.77 (0.66, 0.89)	< 0.001	1.00	0.77 (0.53, 1.12)	0.57 (0.38, 0.86)^**^	0.47 (0.31, 0.70)^***^	< 0.001
Carnitine	0.85 (0.73, 0.98)	0.026	1.00	0.79 (0.56, 1.17)	0.76 (0.52, 1.13)	0.68 (0.45, 1.02)	0.062
**Males**							
TMAO	1.28 (1.07, 1.53)	0.006	1.00	1.29 (0.79, 2.11)	1.03 (0.62, 1.69)	2.08 (1.29, 3.36)^*^	0.009
Choline	1.33 (1.11, 1.59)	0.002	1.00	1.29 (0.78, 2.14)	1.78 (1.09, 2.90)*	1.85 (1.14, 3.02)^*^	0.006
Betaine	0.79 (0.66, 0.94)	0.009	1.00	0.74 (0.47, 1.17)	0.56 (0.35, 0.90)^*^	0.53 (0.33, 0.85)^**^	0.004
Carnitine	0.86 (0.72, 1.03)	0.104	1.00	0.75 (0.48, 1.20)	0.81 (0.50, 1.27)	0.65 (0.40, 1.05)	0.107
**Females**							
TMAO	0.90 (0.69, 1.17)	0.429	1.00	0.77 (0.35, 1.71)	0.66 (0.29, 1.47)	0.84 (0.38, 1.86)	0.624
Choline	1.45 (1.07, 2.00)	0.017	1.00	1.37 (0.57, 3.31)	1.53 (0.66, 3.56)	2.07 (0.92, 4.66)	0.076
Betaine	0.73 (0.57, 0.95)	0.017	1.00	0.88 (0.43, 1.80)	0.63 (0.29, 1.38)	0.30 (0.13, 0.72)^**^	0.005
Carnitine	0.83 (0.63, 1.09)	0.174	1.00	0.89 (0.42, 1.89)	0.68 (0.31, 1.52)	0.79 (0.36, 1.73)	0.444
**≤45 years old**							
TMAO	1.12 (0.89, 1.41)	0.341	1.00	1.11 (0.59, 2.10)	0.92 (0.48, 1.76)	1.61 (0.85, 3.04)	0.256
Choline	1.50 (1.17, 1.94)	0.002	1.00	1.52 (0.76, 3.06)^*^	2.02 (1.01, 4.03)	2.32 (1.16, 4.64)^**^	0.011
Betaine	0.93 (0.73, 1.16)	0.470	1.00	1.62 (0.88, 2.97)	1.03 (0.53, 2.00)	0.88 (0.43, 1.80)	0.500
Carnitine	0.81 (0.64, 1.03)	0.080	1.00	0.86 (0.46, 1.59)	0.63 (0.33, 1.21)	0.61 (0.32, 1.19)	0.093
**>45 years old**							
TMAO	1.19 (0.97, 1.45)	0.092	1.00	1.14 (0.65, 2.01)	0.92 (0.52, 1.63)	1.70 (0.99, 2.92)	0.080
Choline	1.29 (1.06, 1.57)	0.012	1.00	1.22 (0.69, 2.16)	1.53 (0.89, 2.62)^*^	1.79 (1.05, 3.03)^*^	0.022
Betaine	0.68 (0.56, 0.83)	< 0.001	1.00	0.45 (0.27, 0.75)^**^	0.38 (0.23, 0.65)^***^	0.31 (0.19, 0.53)^***^	< 0.001
Carnitine	0.89 (0.74, 1.07)	0.220	1.00	0.75 (0.45, 1.26)	0.86 (0.52, 1.41)	0.76 (0.45, 1.28)	0.391

For the definition of abbreviations, see **Table 1**.

a In according to the quartiles based on TMAO, choline, betaine, and carnitine, separately, TMAO levels for the quartile groups were as follows: Q1 <0.98, Q2: 0.98~1.58, Q3: 1.59~2.52, Q4 > 2.52mmol/L; Choline levels for the quartiles were as follows: Q1 <7.2, Q2: 7.2~8.5, Q3: 8.6~10.1, Q4 > 10.1mmol/L; Betaine levels for the quartiles were as follows: Q1 < 37.3, Q2: 37.3~43.3, Q3: 43.4~51.3, Q4 > 51.3mmol/L. Carnitine levels for the quartiles were as follows: Q1 < 48.2, Q2: 48.2~55.1, Q3: 55.2~61.9, Q4 > 61.9mmol/L.

*Adjusted for traditional risk factors include age, sex, and body mass index; *P < 0.05, **P < 0.01, ***P < 0.001.

### 3.2 Prediabetes and Diabetes-Associated Intestinal Microbial Species and Metabolites

**Figure 2:**
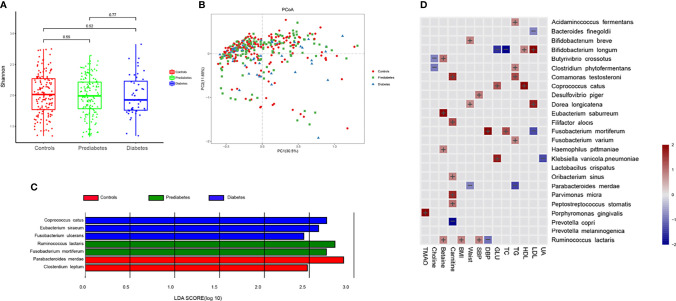
Prediabetes and diabetes-associated intestinal microbiota. **(A)** Box plot showing the species-based α-diversity (Shanon index) in controls, prediabtes, and diabetes. **(B)** Species-based principal coordinates analysis (PCoA) of controls, prediabtes, and diabetes. **(C)** Linear discriminant analysis (LDA) effect size (LEfSe) analysis revealed significant bacterial differences in fecal microbiota in controls, prediabtes, and diabetes. **(D)** Associations between clinical parameters, intestinal microbiota-generated metabolites and microbial species were estimated by MaAsLin2.

We further performed metagenomic sequencing of 361 fecal samples (56 samples from diabetes, 145 from prediabetes, and 160 healthy controls), and the baseline characteristics of individuals were presented in **Table S1**. The shannon index based on the species profile was calculated to estimate the within-sample (α) diversity. The α-diversity of the intestinal microbiome was similar at the species level in the three groups. Similarity, no significant differences were found in β-diversity based on PCoA between the three groups. Genes were aligned to the NR database and annotated to taxonomic groups, and a supervised comparison of the microbiota by utilizing the LEfSe analysis was performed. Our results identified 3 bacterial species consisting *Coprococcus catus*, *Eubacterium siraeum*, and *Fusobacterium ulcerans* were significantly enriched in the diabetes group. Two species *Ruminococcus lactaris* and *Fusobacterium mortiferum*, were enriched in the prediabetes group. Other two bacterial species, including *Parabacteroides merdae* and *Clostridium leptum*, were enriched in the normal glucose control group (**Figures 2A–C**).

Results of multivariate association analysis with MaAsLin2 between microbiota-generated metabolites, clinical indices, and microbial species were presented in **Figure 2D**. Among the microbial species significantly correlated with blood glucose, *Klebsiella variicola.pneumoniae* and *Coprococcus catus* were positively associated, whereas *Bifidobacterium longum* were inversely associated with blood glucose. Physiological parameters of SBP, DBP, BMI, waistline, TC, TG, HDL-C and HDL-C were also included in the analysis. We observed that microbial species enriched in diabetes or prediabetes was generally positively with adverse metabolic parameters, whereas species enriched in controls was associated with improved metabolic parameters, such as *Parabacteroides merdae* was inversely associated with TG and waistline. For microbiota-generated metabolites, *Porphyromonas gingivalis* was positively correlated with TMAO. *Butyrivibrio crossotus* and *Clostridum phytofermentans* were inversely correlated with choline. Four species, including *Butyrivibrio crossotus*, *Eubacterium saburreum, Haemophilus pittmaniae* and *Ruminococcus lactaris* was posively associated with betaine.

### 3.3 Identification of Prediabetes and Diabetes Based on Machine Learning Algorithms

**Figure 3:**
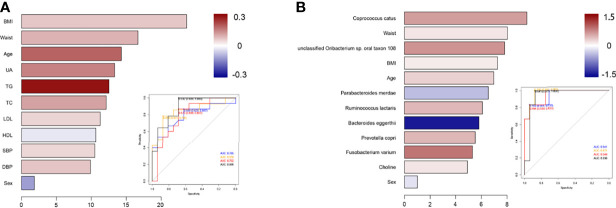
Classification models using selected indicators to identify prediabetes or diabetes patients from controls. **(A)** The selected traditional risk indicators distinguished prediabetes from control based on the Random Forest model. The lengths of bar in the histogram represent Gini coefficient, which indicates the importance of the indicators for classification. The color denotes the enrichment of indicators in control (blue) and in prediabetes or diabetes (red). ROC of classifier models using four groups of biomarkers for prediabetes versus control. AUC = 0.785 for biomarkers 1 (blue curve), AUC = 0.839 for biomarkers 2 (yellow curve), AUC = 0.792 for biomarkers 3 (red curve), and AUC = 0.888 for biomarkers 4 (black curve). **(B)** The ANOVA-selected indicators distinguish diabetes from control based on the Random Forest model. ROC of classifier models using four groups of biomarkers for diabetes versus control. AUC = 0.941 for biomarkers 1 (blue curve), AUC = 0.971 for biomarkers 2 (yellow curve), AUC = 0.948 for biomarkers 3 (red curve), and AUC = 0.938 for biomarkers 4 (black curve).

To illustrate the microbial and metabolic signature of prediabetes and diabetes, we exploit the potential of microbiome and metabolites for classifying prediabetes and diabetes from controls. The strategy of combining classical statistics and multivariate statistics were carried out, and we found the biomarkers distinguishing prediabetes from controls using traditional risk factors, and biomarkers that distinguished diabetes from controls using P value based on one-way ANOVA. Moreover, after 10-fold cross-validation, RF model showed highly promising performance for classifying prediabetes and diabetes from controls (prediabetes vs. controls, diabetes vs. control) (**Figure 3**). For diabetes, compared with models using traditional risk factors (AUC=0.938) or only using microbiome indicators (AUC=0.948), a RF algorithm integrating traditional risk factors with microbiome and metabolites performed better (AUC=0.971). The most informative microbiome features contributing to this classifier were *Coprococcus catus*, *Parabacteroides merdae*, *Ruminococcus lactaris*, *Bacteroides eggerthii*, *Prevotella copri*, and *Fusobacterium varium*, and choline was more effective than TMAO for classifying diabetes from controls (*P* value for Gini coefficient <0.05). To further elucidate whether sex has an effect on the microbial and metabolic signature, we also built models for classifying prediabetes and diabetes from controls by sex (**Figure S2**). For diabetes in males, the most informative microbiome features contributing to this classifier were *Coprococcus catus*, *Fusobacterium varium, Parabacteroides merdae, Ruminococcus lactaris, Prevotella copri* and *Bacteroides eggerthii*., For diabetes in females, the most informative microbiome were *Bacteroides eggerthii, Prevotella copri, Coprococcus catus, Parabacteroides merdae, Fusobacterium varium*, as well as *Ruminococcus lactaris*. The most informative microbiome features contributing to this classifier ranked somewhat differently in males and females. For prediabetes, we observed that the RF model using microbiome and selected traditional risk factors, such as waistline and age, did not display the better predictive performance (AUC=0.839) compared with that only using traditional risk factors (AUC=0.888). After sex stratified, the pattern was consistent, also, the risk factors ranked differently in males and females”. Sensitivity analyses by further adjusted lifestyle risk factors were conducted to inspect the robustness of our findings, and the selected indicator to build the classification models were consistent.

### 3.4 Functional Characterization in Intestinal Microbiome of High or Low Choline Levels

**Figure 4:**
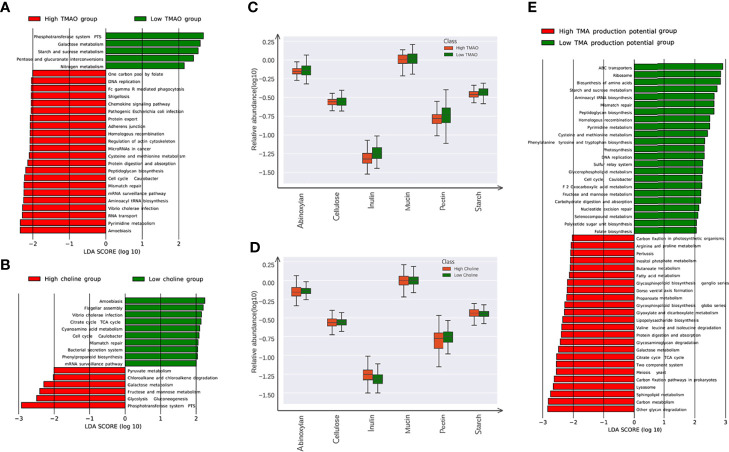
Microbial gene functions annotation in the low (lower thirds) and high (higher thirds) TMAO/Choline groups. **(A)** The average abundance of KEGG modules differentially enriched in gut microbiome of the low and high TMAO groups. Five modules enriched in low TMAO group, and 22 modules overrepresented in high TMAO group are shown in green and red, respectively. **(B)** The average abundance of KEGG modules differentially enriched in gut microbiome of low and high choline groups. Five modules enriched in low choline group, and twenty twou modules overrepresented in high choline group are shown in green and red, respectively. **(C)** The average abundance of CAZy family involved in metabolism of inulin, pectin, and starch significantly altered in the low and high TMAO groups. **(D)** The average abundance of CAZy family involved in metabolism of inulin significantly altered in the low and high choline groups. **(E)** The average abundance of KEGG modules differentially enriched in gut microbiome of groups with the low and high TMA production potential. 23 modules enriched in low TMA production potential group, and 24 modules overrepresented in high TMAO group are shown in green and red, respectively.

All the genes were aligned to the KEGG database and CAZy database, and proteins were assigned to the KEGG orthology and CAZy families. Pathways involved in carbohydrate metabolism were enriched in high choline or low TMAO group. KEGG pathways including ‘glycolysis gluconeogenesis’, ‘fructose and mannose metabolism’, and ‘galactose metabolism’, were all highly enriched in the microbiome of high choline individuals. Conversely, KEGG pathways belonging to the ‘pentose and glucoronate interconversions’, ‘starch and sucrose metabolism’, and ‘galactose metabolism’, were significantly enriched in the microbiome of low TMAO individuals (**Figures 4A, B**). Among the CAZy genes for metabolizing different carbohydrate substrates, those contributing to insulin degradation were significantly enriched in high choline group, whereas those contributing to starch, insulin and pectin degradation were enriched in low TMAO group (**Figures 4C, D**). According to the TMA production potential, TMAO production potential was inversely correlated to glucose level, although the association did not reach statistical significance. Several KOs associated with the carbohydrate metabolism process were enhanced in individuals with low TMA production potential (**Figure 4E**).

We next examined FishTaco’s calculated taxon-level contributions to functional differences, focusing on carbohydrate metabolism pathways, which were observed as choline-associated functional shifts. The specie *Ruminococcus lactaris*, as well as *Coprococcus catus* and *Prevotella copri*, were the main drivers of the enrichment of the carbohydrate metabolism pathway in the condition of high choline. The species *Parabacteroides merdae*, was the major driver of the enrichment in the carbohydrate metabolism pathway, while *Bacteroides eggerthii* attenuated that enrichment in the condition of low choline. At the module level, some species, for example *Prevotella copri*, drove the observed shift in one function while attenuating the shift in another (**Figure S3**).

## 4 Discussion

Our study systematically investigated the associations of intestinal microbiota and microbiota-generated metabolites with glucose metabolism. In this study, we observed that plasma choline was positively, while betaine was negatively associated with diabetes, independently of TMAO in Chinese adults. Individuals with diabetes could be accurately distinguished from controls by integrating data on choline, and certain species abundance, as well as some traditional risk factors such as age, sex, BMI and waistline. Additionally, some species, for example diabetes-associated species *Prevotella copri* drove the observed shift in one function while attenuating the shift in another at the module level, which implies species often had complex impacts on the observed shift in function. Greater attention should be paid to plasma choline because it is more stable, and links the microbiota to impaired glucose metabolism and diabetes.

According to recent series of researches, intestinal microbiota can metabolize trimethylamine (TMA)-containing nutrients to produce TMA in the intestine, which is subsequently converted into TMAO by host FMO3 in the liver (18). Manipulation of TMAO concentrations in mice through inhibiting host FMO3 can prevent the development of hyperglycemia, hyperlipidemia, and atherosclerosis in a diabetic mouse model (10). Plasma level of TMAO was found to be higher in diabetic individuals in observed studies (11, 19). The meta-analysis of continuous variable documented that levels of TMAO were 0.36μmol/L higher in patients with diabetes than in that without diabetes (20). However, in intervention studies, a reduction of choline rather than TMAO showed significant associated with losses of body fat, fasting insulin and HOMA-IR, as well as 2-year improvements in glucose and insulin resistance (12, 21). Similarly, circulating level of choline decreased in morbidly obese patients after bariatric surgery along with level of TMAO significantly increased after the weight loss. Mice fed a choline-deficient diet also observed to have improved insulin resistance and glucose metabolism (22). In our results, there was a positive relationship between plasma choline and adverse glucose metabolism independently of TMAO. We speculate the blood glucose modulated by choline was possibly through different diabetes-related mechanisms besides TMAO. Choline (or the choline metabolite betaine) is a methyl donor involved in one-carbon metabolism and play a critical role in methylation reactions, including DNA methylation, as well as DNA stability and repair. Disruption of epigenetic mechanisms may significantly impact the development of metabolic disease by increasing oxidative stress, reducing chromosome stability, and promoting the development of obesity, insulin resistance, and vascular dysfunction (23). Previous epidemiological study have demonstrated that DNA methylomic changes are associated with chronic health conditions such as glucose level alteration, and most DNA meta-methylome changes occurred 80-90 days before clinically detectable glucose elevation (24). Besides, data from KEGG pathways and Cazy enzymes showed microbial functions in the condition of high choline displayed higher capacity for carbohydrate utilization, by which we also speculated that microbiota might directly induce adverse glucose metabolism through other metabolites, rather than TMAO production. For example, intestinal microbiota was able to synthesize amino acids, such as aromatic amino acids (AAAs) and branched-chain amino acids (BCAAs), and choline was further positively connected to these diabetes-related amino acids (12, 25).

Plasma betaine, contrary to choline, was inversely associated with diabetes in our study. Previous study has showed that plasma choline and betaine were investigated in relation to cardiovascular disease risk with opposite directions, that choline was positively, conversely betaine was inversely associated with several components of cardiometabolic risk profiles in different populations (26, 27). Glycine betaine mainly from the food items could be transformed into a group of betainized compounds by the gut microbiota. In recent interventional and animal studies, betainized compounds correlated with improved glucose metabolism and the risk of diabetes (28). Among adults with the metabolic syndrome, PAB, one betainized compounds, was associated with favorable fasting insulin, lipid profiles and inflammation (29). Several bacterial taxa, including *Akkermansia*, *Bifidobacterium*, *Coriobacteriaceae*, *Lactobacillus*, *Parasutterella*, and *Ruminococcus*, may involve in betaine metabolism in animal study (30). Betaine is formed in kidney and liver by choline oxidation, or obtained from food of cereal grains, especially whole-grain rye and wheat. Betaine serves as a methyl donor in the betaine-homocysteine methyltransferase reaction, which is responsible for the betaine-dependent remethylation of homocysteine to methionine (31). There is an important crosstalk between choline/1-carbon metabolism (such as betaine) and the pathways of insulin sensitivity, fact deposition and energy metabolism through epigenetic modifications. This may explain why there is a paradox: increased plasma concentration of choline associated with hyperglycemia, but decreased plasma concentration of betaine also related to hyperglycemia. Given that most of the evidence is cross-sectional, it cannot be used to establish cause and effect between betaine deficiency and hyperglycemia. Diagnostic performance of betained compounds in blood is important for future research, which need further studies to elucidate mechanisms.

Two independent metagenome-wide association studies in European and Asian patients with diabetes, showed that the concentrations of butyrate-producing such as *Roseburia intestinalis* and *Faecalibacterium prausnitzii* decreased in diabetic subjects, and the proportion of opportunistically pathogenic *Clostridium* species increased (2, 32). Zhang et al. (33) focused on the analysis of the intestinal microbiota in prediabetes using 16S rDNA-based high-throughput sequencing. Patients with prediabetes already differed from normal glucose people, that prediabetes had lower proportions of butyrate-producing bacteria such as *Akkermansia muciniphila ATCCBAA-835*, and *Faecalibacterium prausnitzii L2-6*, whereas bacteria such as *Clostridiales* sp. *SS3/4*, and *Haemophilus parainfluenzae T3T1* were more abundant. In Danish adults, the intestinal microbiota differed the most between prediabetes and controls were genus *Clostridium* and *Akkermansia muciniphila*, which both displayed lower abundance in prediabetes group (34). Despite there was a similar trend in some universal butyrate-producing bacteria, the bacterial taxa were markedly different in diverse populations, demonstrating the microbiota is dramatically impacted by research method, diet, medication use, ethnicity and geographical locations. In our study, we found that *Coprococcus catus* belonged to *Firmicutes* was the common microbial characteristics of diabetes, and contributed a lot to distinguish individuals with diabetes from controls, which is consistent with previous studies that phyla *Firmicutes* (eg.*Coprococcus catus*) were more abundant in obese people those had worse glucose level (35, 36). *Ruminococcus* flora help gut epithelial cells to absorb sugars, which might contribute to weight gain in the host. Results from diabetic rats model demonstrated that blood glucose was positively correlated with *Ruminococcus (* 37). In previous animal studies, two *Parabacteroides* species, including *Parabacteroides distasonis* and *Parabacteroides goldsteinii*, played roles in anti-obesity, hyperglycemia, and insulin resistant (38, 39). *Parabacteroides merdae*, which is opportunistic pathogenic taxa, was reported frequently distributed in hypertensive gut microbiome, further investigations whether *Parabacteroides merdae* play preventive role in the progression of diabetes need be conducted. Since a number of *Ruminococcus* species are known to be associated with metabolic diseases, also *Bacteroides eggerthii* abundance was reported significantly higher in obese children and correlated positively with body fat percentage and negatively with insoluble fiber intake (40). Based on data from the MetaHIT, *Prevotella copri* was identified as the main species driving the positive association between biosynthesis of branched-chain amino acids (BCAAs) and insulin resistance, and mice fed with *Prevotella copri* had increased insulin resistance, aggravate glucose intolerance and elevated levels of BCAAs (41). Deficiency of gut short-chain fatty acid (SCFA) is associated with diabetes. We also observed that several *Bifidobacterium* species, including *Bifidobacterium bifidum*, *Bifidobacterium_longum*, *Bifidobacterium_breve*, and *Bifidobacterium adolescentis* were important for classification prediabets and control groups. Zhao et al. (42) found that adopting a high-fiber diet promoted the growth of SCFA-producing species including *Bifidobacterium longum* in diabetic humans, in company with the elevated levels of glucagon-like peptide-1, a decline in hemoglobin A1c levels, and improved blood glucose levels. Although the results yielded different species biomarkers, our findings indicated that choline may be more effective to classify diabetes group from control group, comparing to specific species.

Our study has several strengths and limitations. First, this study introduced data of metagenome and microbiota-generated metabolites in a population-based study, which is to date the largest in Asian allowing us to directly examine bacterial functional genes. Second, we enrolled relatively healthy study population, whereas many of the TMAO studies to date have been limited to participants with metabolic disease or undergoing various medical procedures. Third, we were also able to assess associations of disease biomarkers not only with TMAO, but also choline, carnitine, and betaine. Our study also had some limitations. The major limitation of this study was a cross-sectional study, and the statistically significant association between intestinal microbiota, microbiota-generated metabolites and host health do not establish causality. Longitudinal studies are needed to determine the stronger evidence of these associations. Second, we did not have postprandial blood sugar or HbA1c data available in this study, which may induce a misclassification bias. However, the misclassification of undiagnosed diabetic women into normal glycemic group was more likely to weaken the associations. Third, we did not adjust for potential confounders such as dietary factors. Diet may modify the associations of choline or its metabolites with disease risk. However, in the sensitive analyses, the results were somewhat consistent after further adjusted lifestyle factors including dietary habit.

In this study, we found that the integrating choline and microbiota species, as well as traditional risk factors specific was consistently more effective to classify diabetes from healthy controls. The blood glucose metabolism modulated by microbial metabolites, such as choline and TMAO, were possibly through different diabetes-related mechanisms. These results provide evidence that higher microbial choline was positively associated with glucose metabolism and type 2 diabetes, especially highlight great potential ability for classifying diabetes population by choline and specific intestinal species.

## Data Availability Statement

The datasets presented in this study can be found in online repositories. The names of the repository/repositories and accession number(s) can be found below: https://www.ncbi.nlm.nih.gov/, PRJNA511714.

## Ethics Statement

The studies involving human participants were reviewed and approved by Ethical Committee of the Chinese People’s Liberation Army General Hospital. The patients/participants provided their written informed consent to participate in this study.

## Author Contributions

Conceptualization: QZ, LZ, YH, and MZ. Data curation: QZ, YH, XZ, MZ. Formal analysis: All authors. Funding acquisition: QZ, XZ, YH. Investigation: All authors. Methodology: QZ, YH, XZ, MZ. Project administration: QZ, XZ. Resources: QZ, XZ. Software: YH, XC, DL. Supervision: QZ, YH. Validation: QZ, XZ. Writing - original draft: QZ, MZ, FW. Writing - review and editing: YH, HL, XZ, YPL. QZ, LZ and YH are the guarantors of this work and, as such, had full access to all of the data in the study and take responsibility for the integrity of the data and the accuracy of the data analysis. All authors contributed to the article and approved the submitted version.

## Funding

This work was supported by Grants 81872920, 81602854, 81561128020, 91639108, 81370235 and 81970425 from the Natural Science Foundation of China. This project was also supported by Grant 2016YFC0903000 from Ministry of Science and Technology of China.

## Conflict of Interest

Author XC was employed by the company Bao Feng Key Laboratory of Genetics and Metabolism in Beijing. Author DL was employed by the company WeHealthGene Institute in Shenzhen.

The remaining authors declare that the research was conducted in the absence of any commercial or financial relationships that could be construed as a potential conflict of interest.

## Publisher’s Note

All claims expressed in this article are solely those of the authors and do not necessarily represent those of their affiliated organizations, or those of the publisher, the editors and the reviewers. Any product that may be evaluated in this article, or claim that may be made by its manufacturer, is not guaranteed or endorsed by the publisher.

## Abbreviations

AUC, Area under the receiver operating curve; CAZy, Carbohydrate-Active EnZymes; CVD, Cardiovascular disease; FMO, Flavin monooxygenase; HDL, High-density lipoprotein; HOMA-IR, Homeostasis model assessment of insulin resistance; IQR, Interquartile range; KEGG, Kyoto Encyclopedia of Genes and Genomes; KO, KEGG orthologue group; LDL, Low-density lipoprotein; LEfSe Linear discriminant analysis (LDA) effect size; MRM, Multiple reaction monitoring; ROC, Receiver operating characteristic; TC, Total cholesterol; TG, Triglyceride; TMA, Trimethylamine; TMAO, Trimethylamine-N-oxide; UA, Uric acid.
