# Supplementary material for: Integrating Choline and Specific Intestinal Microbiota to Classify Type 2 Diabetes in Adults: A Machine Learning Based Metagenomics Study
Source: Front Endocrinol (Lausanne). 2022 Jun 27;13:906310. doi: 10.3389/fendo.2022.906310 (PMC9271784; doi:10.3389/fendo.2022.906310)
Supplement: Supplementary file 1 [file DataSheet_1.docx]

Supplementary Material


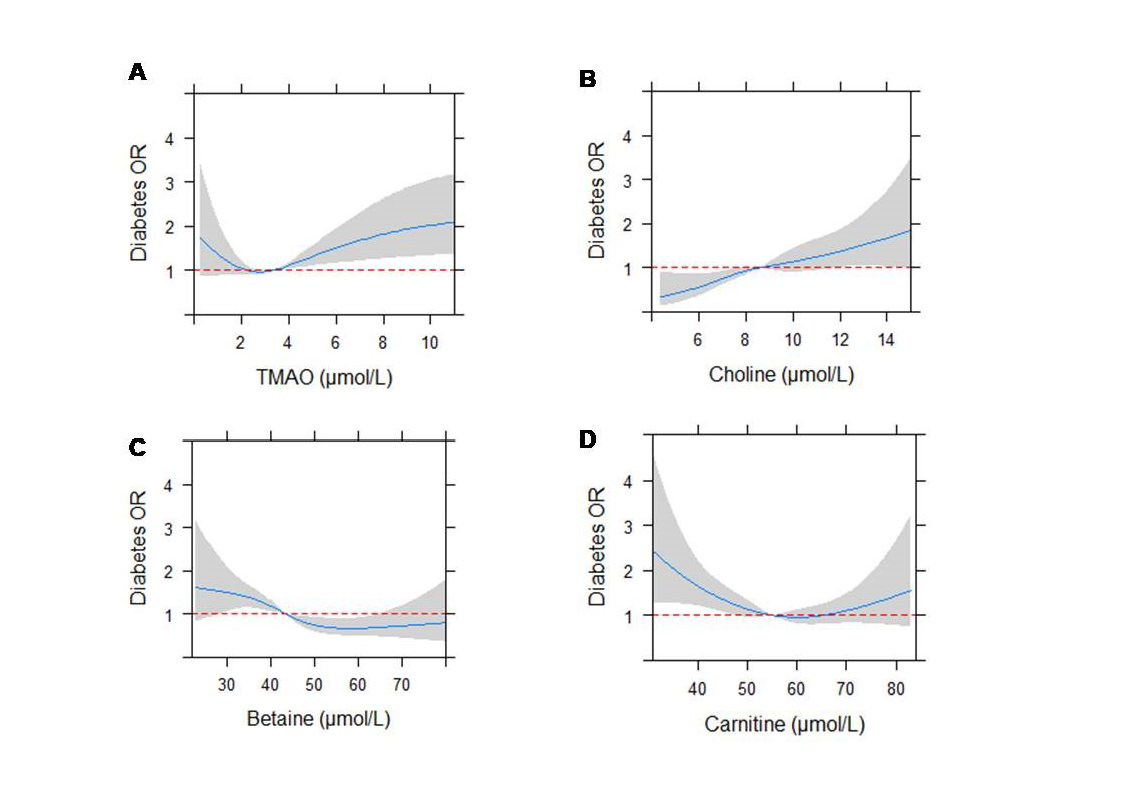


**Supplementary Figure 1.** The concentration-response curves for TMAO (A), choline (B), betaine (C) and (D) carnitine concentrations on diabetes. OR indicates odds ratio.


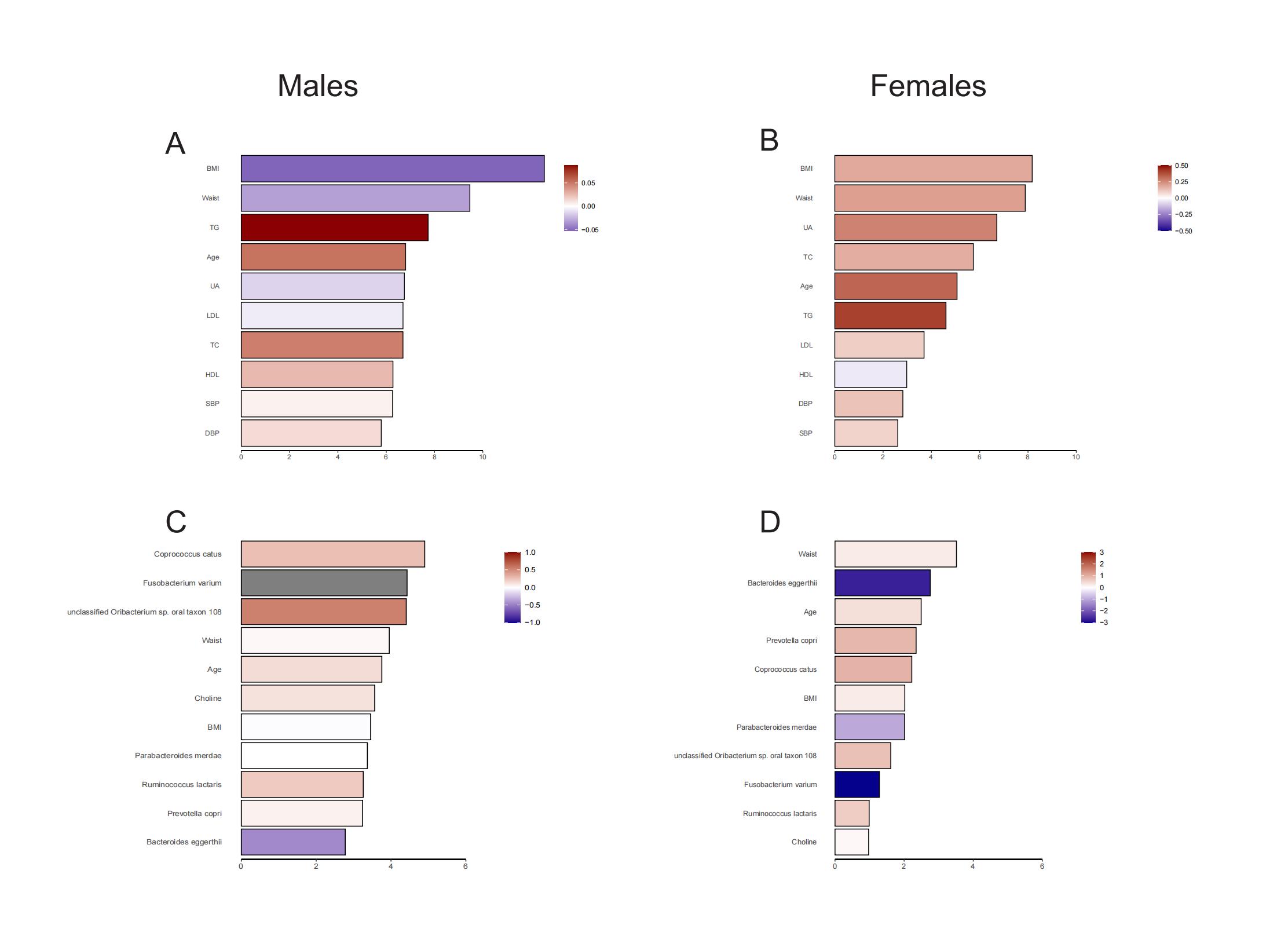


**Supplementary Figure 2.** Classification models using selected indicators to identify prediabetes or diabetes patients from controls by sex. (A) The selected traditional risk indicators distinguished prediabetes from control in males based on the Random Forest model. The lengths of bar in the histogram represent Gini coefficient, which indicates the importance of the indicators for classification. The color denotes the enrichment of indicators in control (blue) and in prediabetes or diabetes (red). (B) The selected traditional risk indicators distinguished prediabetes from control in females based on the Random Forest model. (C) The ANOVA-selected indicators distinguish diabetes from control in males based on the Random Forest model. (D) The ANOVA-selected indicators distinguish diabetes from control in females based on the Random Forest model.


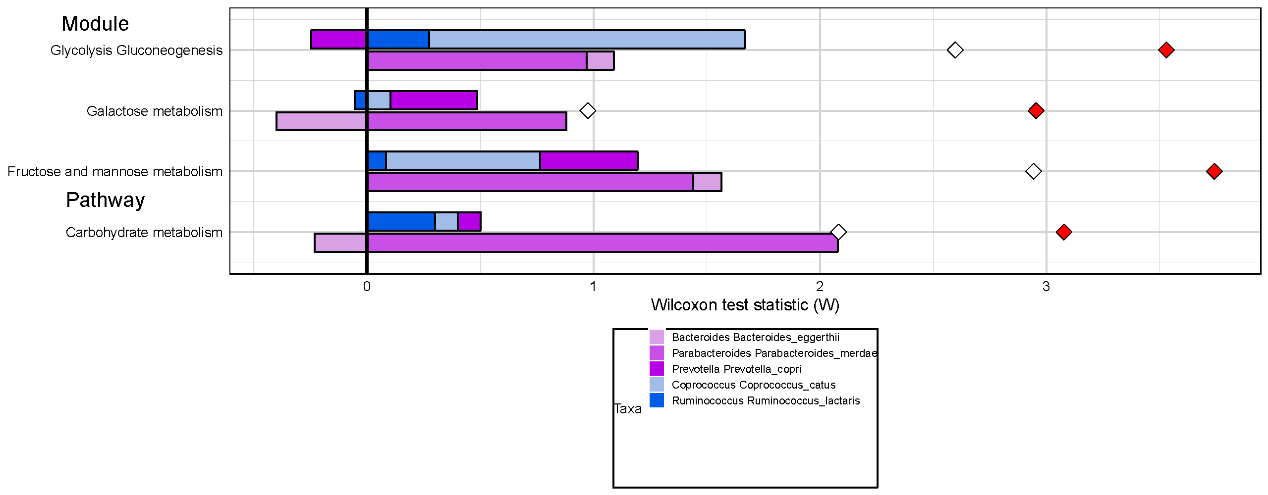


**Supplementary Figure 3.** Identifying the Taxonomic Contributors in the low (lower thirds) and high (higher thirds) Choline groups.
